# Oral Health in Zero Gravity: A Comprehensive Review of Orofacial Effects and Countermeasures in Spaceflights

**DOI:** 10.7759/cureus.49035

**Published:** 2023-11-19

**Authors:** Divya Harika Pedada, Karan Hiral Mehta, Saraswathi Sravani Pulluri, Priyanka Rana, Hanmandla Rajani, Ayesha Aiman

**Affiliations:** 1 Oral Medicine and Radiology, Panineeya Institute of Dental Sciences & Research Centre, Hyderabad, IND; 2 Oral Medicine and Radiology, Government Dental College Kadapa, Kadapa, IND; 3 Health Information Technology, University of Maryland, Baltimore County, Baltimore, USA; 4 Oral Medicine and Radiology, Postgraduate Institute of Medical Education and Research, Chandigarh, IND

**Keywords:** aerospace dentistry, oral health, preventive dentistry, space-adapted dental technologies, space flight, zero gravity

## Abstract

Space is a complex and challenging setting encompassing the region beyond Earth's atmosphere where astronauts and spacecraft operate. The unique conditions of spaceflights, particularly microgravity and radiation, pose significant challenges to astronaut health, including the orofacial region. It has effects on saliva production, microbial composition, and oral hygiene practices, which influence oral health status, such as increased risk of dental caries, gum diseases, oral discomfort, temporomandibular joint dysfunctions, sialoliths, pain and dysesthesia in the teeth and oral mucosa, masticatory muscle atrophy, and oral cancer which can be detrimental during prolonged missions. Hence, a comprehensive approach to dental care in space is imperative to ensure astronauts' well-being and overall health as we strive to extend our presence beyond Earth. This literature review paper sheds light on the intricate effects of space on the orofacial region and delves into the unique challenges astronauts face in upholding optimal oral health while in space. It explores the current state of dentistry in space and discusses advancements and strategies that aim to maintain optimal oral health for astronauts during extended space missions.

## Introduction and background

Man has always had astral dreams when gazing at the night sky. Outer space, usually referred to simply as space, is the boundless region that exists beyond Earth's atmosphere. While there is no visible boundary between the Earth's atmosphere and space, it is widely accepted that space begins 100km above mean sea level [1]. Space exploration became a reality in the second half of the 20th century thanks to the development of rockets with the strength to defy gravity and reach orbital velocity. Modern space exploration is traversing areas that were once beyond the realm of imagination.

In recent years, visits to space have become increasingly frequent, and long-duration space travels are no longer unusual. Differences in numerous parameters between space and Earth, such as microgravity (µG), altered radiation levels and atmospheric pressure, varying temperature, and differing densities in space, trigger a cascade of physical and physiological changes.

Microgravity, a hallmark of space, results in dramatic redistribution of fluids and musculoskeletal alterations, affecting astronauts' overall physiology. Vestibular and sensorimotor systems also experience shifts, requiring adaptation for optimal functioning in a weightless environment. The psychological effects on astronauts, altered flora, and immunodeficiency cannot be overlooked [2,3].

The oral and maxillofacial region is not immune to the effects of space travel. Studies have reported incidences of dental cavities, periodontal diseases, oro-maxillofacial fractures, pain, sensory abnormalities in the teeth and oral mucosa, salivary gland stone formation (sialoliths), craniomandibular joint disorders, masticatory muscle atrophy, and oral cancer in astronauts [4-6]. Evaluating, preventing, and managing these effects in the oral cavity is essential to improve astronauts' quality of life during and after a space flight.

Adapting to physical and physiological changes is a challenge astronauts face during a space flight. To mitigate these physiologic alterations, numerous countermeasures are being developed and tested. Aeronautical dentistry is a newly recognized specialty that deals with the study of dental aspects in an aeronautical environment [6]. This paper delves into the multifaceted effects of space travel on the orofacial region, offering insights into the countermeasures and developments designed to mitigate these effects.

Methodology 

An extensive search was performed utilizing MEDLINE, PubMed, and the Google Scholar databases. We employed a range of keywords such as "aerospace," "microgravity," "space flight," "space mission," "zero-gravity," "oral health," "dentistry," " countermeasures," "preventive measures," and space-adapted dental technologies," to retrieve relevant literature. This review included literature obtained from these electronic searches, as well as related bibliographic entries of those articles.

## Review

The need for aerospace dentistry: addressing reported dental emergencies in spaceflights

Although dental incidents have been minimal in human spaceflights, the possibility of dental emergencies in space escalates with extended spaceflight duration. These challenges, if left unaddressed, could jeopardize the well-being of astronauts during prolonged space missions and the success of the mission itself. Historical data shed light on the dental emergencies of various space missions. During the Apollo-17 program conducted in 1972, no dental problems impacted the 18-day mission. However, five Apollo astronauts among the thirty-three required dental intervention during the three-month preflight preparation. One pulpitis case was seen before and after a space flight [7]. Yuri Romanenko, a Russian cosmonaut, reportedly suffered from a toothache during the 96-day Salyut-6 journey in 1978 with another two weeks of the mission remaining. The only dental advice provided to him was to "take a mouthwash and keep warm" because the Soviet program regrettably had no "contingency plans for dental emergencies." Within the span from March 1995 to June 1998, the Russian Space Station MIR also experienced a dental incident, accounting for approximately 1% of all observed medical occurrences. Specifically, dental caries was diagnosed and managed by applying a temporary dental filling from the station’s dental kit. Furthermore, the MIR project logged 304 medical incidents between February 7, 1987, and February 9, 1996. Among these cases, only one was associated with a case of dental caries, resulting in a 0.01% per 100-day incidence rate [7,8]. In 1977, Brown et al. calculated a 0.92 percent risk for an in-flight dental event that could significantly impair a crew member's productivity [9]. Current data from the Integrated Medical Model (IMM) estimate dental cavities and abscesses, pulpal exposure, tooth avulsion, dental crown replacement, and dental filling replacement as the most likely medical conditions to end in evacuation from the International Space Station [8].

The evolution of aerospace dentistry

The history of aerospace dentistry is a fascinating journey that parallels the advancements in aviation and space exploration. The genesis of dental guidelines within space medicine can be traced back to 1957 when the general office drafted the initial principles for dentistry in the U.S. Air Force manual. The year 1960 marked a pivotal moment with the initiation of an astronautical dental training program, signifying a recognition of the unique challenges posed by dental health in space. In 1966, the Air Force appointed Major William Frome as a dental officer for an all-day role at Houston's National Aeronautics and Space Administration (NASA), responsible for ensuring astronauts' oral health. NASA assigned Colonel Johan Young in 1980 to design dentistry equipment and protocols for use in the unique conditions of zero gravity. In 2000, the Committee on Space Medicine at the National Academy of Science Institute of Medicine deliberated on the methods to preserve astronauts' dental health during prolonged missions. In 2006, at the Aerospace Medical Association meeting in Orlando, Florida, Dr. Leon Dychter proposed the idea of forming the “International Association of Aerospace Dentistry.” In 2008, IAAD was established. Dr. Balwant Rai was appointed the Mars Desert Research Station's dental health and safety officer in 2009 [6]. Ongoing research in aerospace dentistry focuses on developing innovative technologies and approaches to address oral health challenges associated with long-duration space missions, lunar bases, and future Mars missions. Studies are conducted either in spaceflights or in a simulated microgravity environment.

Spaceflight Experiments

Spaceflight experiments are a valuable method for studying microgravity-induced changes, but they have limitations. Infrequent mission launches limit opportunities. The experimental setup needs to abide by shipment weight and space limitations, leading to small sample sizes and minimal biological replicates. It is essential to have well-built machinery parts to ensure that experimental models can withstand the challenging conditions during launch, acquisition of data, and Earth return. The strategies to overcome these limitations ultimately make these spaceflight experiments costly, leading to higher financial burdens. As a result, many researchers opt for simulated microgravity on Earth to study microgravity-induced changes [10].

Simulated Microgravity Techniques

Horizontal bed rest studies were initially used to mimic humans in weightlessness but failed to replicate fluid redistribution towards the head. Overtime Researchers started conducting experiments in the head-down tilt (HDT) position by inclining subjects towards the head to encourage the shift of bodily fluids away from the lower extremities toward the head. The degree of tilt in the HDT position varies from 4° to 15°, with a 6° tilt angle being homologous for microgravity simulation in most human bed rest studies. While HBT/HDT minimizes microgravity forces, water immersion, used since the 1960s for astronaut preparation, acts through neutral buoyancy. Water immersion in microgravity simulation involves a subject sitting in water at 34-35°C. However, prolonged immersion can cause adverse cutaneous effects, leading to dry immersion's introduction. Dry immersion is a technique in which individuals are submerged in a bath up to the neck level while remaining completely dry with a waterproof, highly flexible fabric. This neutral buoyancy concept prevents localized pressure at the seat and feet, allowing more extended experiments for up to 56 days [10].

Spaceflight-induced changes in the oral and maxillofacial region

Bone Changes

A profound biological effect of human spaceflight is bone loss and alterations in the skeleton caused by microgravity. Disuse atrophy due to exposure to microgravity leads astronauts to lose calcium from their bones, similar to osteoporosis. The four main types of bone cells, osteoblasts, osteocytes, osteoclasts, and osteogenic cells, are affected by microgravity. The ability of osteoblasts to proliferate, differentiate, and react to stimuli is diminished, leading to bone loss. Researchers believe bone deposition and resorption changes due to the reduced blood and plasma volume experienced during a space flight [5].

In spaceflights, one can observe osteoporosis of the maxilla and mandible. NASA astronauts' bone mineral loss during a space flight has been studied for over 40 years. An estimated 1% to 2% of bone mass is lost monthly. Bones lose their mass differently depending on the location and the mechanical pressure they are subjected to [11]. It is a medical requirement at Jhonson Space Centre (JSC) to monitor changes in bone mass before and after the flight. Sibonga et al. conducted a study in JSC and evaluated the medical data of 45 crew members, including cosmonauts, to track the recovery of the lost bone minerals. The analysis plotted changes in bone mineral density (BMD) as a function of time and estimated the 50% recovery time in various areas. The results suggest that bone mineral loss during a space flight is a significant issue [12]. Caillot-Augusseau et al. conducted a study on a 36-year-old cosmonaut before, during, and after a 180-day space flight, showing that microgravity caused an uncoupling of bone remodeling between formation and resorption, potentially accounting for bone loss [13]. Rai et al. recorded a significant decrease in bone and mineral density of alveolar bone and elevated levels of matrix metalloproteinases MMP-8 and MMP9 cathepsin k, and osteocalcin levels in gingival crevicular fluids in simulated microgravity conditions [14].

Salivary Changes

Various studies suggested salivary changes in the microgravity environment. Sun examined the effects of microgravity on oral salivary secretory function and the microbiome in a simulated microgravity environment for 15 days and observed no significant pH, flow rate, or alpha diversity differences but noticed an increase in oral disease-related bacteria and decreased oral health-related commensal bacteria [15]. However, Rai et al. concluded that flow rate, sodium, potassium, calcium, phosphate, and protein levels were increased in microgravity simulation environments compared to normal, and the same findings were observed in urine. It was also mentioned that environmental and dietary factors may adversely affect salivary composition and increase salivary stone formation risk during a space flight [16]. The decrease in stimulated salivary secretion might also be a result of reduced functionality in the muscles responsible for the salivary gland. Elevated bone resorption in microgravity conditions leads to the deposition of calcium salts, specifically calcium oxalate and calcium phosphate, in the saliva, forming salivary stones [17]. Saliva is also an emerging non-invasive diagnostic aid. Studies are being carried out using salvia as a diagnostic aid in aerospace. During long-duration missions, the International Space Station (ISS) crew's saliva samples reveal elevated levels of antimicrobial proteins, including IgA, lysozyme, LL-37, and cortisol-to-dehydroepiandrosterone [18].

Microbiome Changes

Culture investigations performed during Skylab missions indicate that oral microbial populations may change during a space flight, as increased counts of intra-oral anaerobic bacteria were documented in the in-flight samples compared to the pre-flight samples collected from the 18 astronauts that were being investigated. Streptococcus was the most numerous bacteria in the saliva, accounting for 8% of all the species found, and their diversity reduced throughout spaceflight. Other species that changed statistically were Proteobacteria and Fusobacteria, which increased during flight, and Actinobacteria, which decreased. Catonella, Megasphera, and Actinobacillus were absent in more than half of the pre-flight saliva samples but were discovered during flight. The relative abundances of these taxa increased during flight in participants who previously had them at pre-flight [19]. During space missions involving the space shuttle, Russian Soyuz, and the ISS, there have been documented cases of latent herpes viruses reactivating in astronauts. Reactivation of these viruses has also been noted in terrestrial experiments simulating spaceflight conditions, such as those conducted in Antarctica, undersea habitats, experiments with artificial gravity, and studies involving extended bed rest. Of 89 astronauts who participated in short-duration space shuttle flights, approximately 53% showed evidence of one or more herpes viruses in their saliva or urine samples. Notably, the extent and frequency of viral shedding were directly linked to the duration of their spaceflights, with significant reactivations of Epstein-Barr virus (EBV), cytomegalovirus (CMV), and varicella-zoster virus (VZV) occurring while in the space. Specifically, the percentage of VZV shedding increased from 41% during space shuttle missions to 65% during ISS missions, while EBV shedding increased from 82% to 96%. CMV, known for its immuno-suppressive properties, might contribute to observed immune dysfunction in crew members [20].

Altered Immunity

The microgravity environment and factors such as physiological stress, isolation, radiation, and disrupted circadian rhythm can compromise the human immune system. Numerous inflight studies have shown differences in the distribution of WBCs, a decline in the effectiveness of T cells, and shifts in the production of cytokines. Changes in immunity are noticed in brief period LEO (low earth orbit) missions. For extended spaceflights, these persistent changes in the immune system might also lead to unfavorable outcomes, including the possibility of malignant diseases [21].

*Masticatory Muscle Atrophy * 

The reduction in the usage of antigravity muscles in a weightless environment causes muscles to weaken and deteriorate. The masseter, temporalis, and medial pterygoid are the mandible-elevating muscles that function against gravitational forces and hence are more susceptible to atrophy. Phillippou et al. compared the responses of masticatory and appendicular muscles to microgravity using mice aboard the Space Shuttle Space Transportation System-135. Results suggested that the tibialis anterior muscle showed a decreased mass, decreased phosphorylated (P)-Akt, and increased atrogene expression after 13 days of space flight. In contrast, Masseter exhibited no notable alterations in mass, but there is an increase in P-focal adhesion kinase, P-Akt, and expression of atrogene. This suggested that distinct muscles showed different set points of adaptation, with the masticatory muscle being more stable than the tibialis anterior [22].

 *Decompression Sickness (DCS)*

Nitrogen dissolved in tissues, blood, and joints can be released as microbubbles as the pressure decreases during a space flight, causing DCS. It is a condition where gas emboli are formed in tissues due to a rapid decrease in the surrounding air or water pressure, causing compression of surrounding nerves and blood vessels. Boyle's law and Henry's law explain this phenomenon. DCS may cause skin changes and milder symptoms, such as joint pain, and more severe symptoms involving the neurologic, cardiac, and pulmonary systems. Several factors can heighten the likelihood of experiencing decompression sickness during low-pressure conditions, including physical exertion and individual vulnerability. Prior to the extravehicular activity, astronauts undergo a process of breathing pure oxygen to release the body’s stored nitrogen and diminish the risk of DCS [23].

Free Radical Activity

On Earth, the typical annual radiation exposure for an individual is under 0.005 sievert (sv), whereas on the International Space Station, it amounts to approximately 0.3sv per year [4]. The primary sources of ionizing space radiation are the enclosed radiation zones, interstellar cosmic rays, and solar flare particle events. The Earth's magnetosphere protects significantly from ionizing radiation in near-Earth orbit, but this safeguard is absent during interplanetary journeys [20]. Reduced concentrations of vitamins E and C and elevated levels of malonaldehyde denote an upsurge in free radical activity in space missions [4]. Exposure to gaseous and particulate toxins resulting from human metabolic processes, debris and garbage, spacecraft chemicals, propulsion agents, heat dissipators, and emissions from plastics significantly increases the risk of developing cancers. The currently acknowledged safety threshold for astronauts is a restriction that space activities should not result in more than a 3% elevated risk of cancer-related mortality throughout their careers [21].

Changes in Drug Metabolism

Medication during a space flight may not be as effective as expected in managing medical concerns on Earth. Drug pharmacological efficacy can change due to alterations in bodily function and metabolism in space. Crew members expressed dissatisfaction with the effectiveness of certain medications in addressing common issues during expeditions [24]. In 1999, Putcha et al. reported instances of medications being ineffective during Space Shuttle flights. They noted 13 different drugs crew members described as "not effective" or "mildly effective" in addressing their symptoms. These medications included oxymetazoline, zolpidem, flurazepam, aspirin, promethazine, temazepam, pseudoephedrine, and acetaminophen [25]. In 2014, Barger et al. discussed the use of sleep-inducing drugs during the Space Shuttle and ISS missions. According to their findings, crew members, in 17-19% of instances, resorted to taking a second dose of medication on the same night when crew members used sleep medication (zolpidem or zaleplon) [26]. Idkaidek et al. conducted a study on paracetamol (APAP) pharmacokinetics during an air flight in 20 healthy volunteers. Saliva samples were collected every 15 minutes up to two hours after dosing. The results showed similar absorption and early exposure rates up to one hour but lower bioavailability after one hour on-board. This could be due to increased liver blood flow at high altitudes and increased liver metabolism [27]. Many drugs taken in space are administered orally, and any changes in absorption rate may lead to changes in drug exposure over time, which may influence treatment efficacy. A variety of enzymes are altered by spaceflight and simulated microgravity. These modifications may result in variations in medication metabolism, which may increase or reduce drug exposure during a space flight, allowing for drug buildup in some situations [28].

Spaceflight-associated oral issues

**Figure 1:**
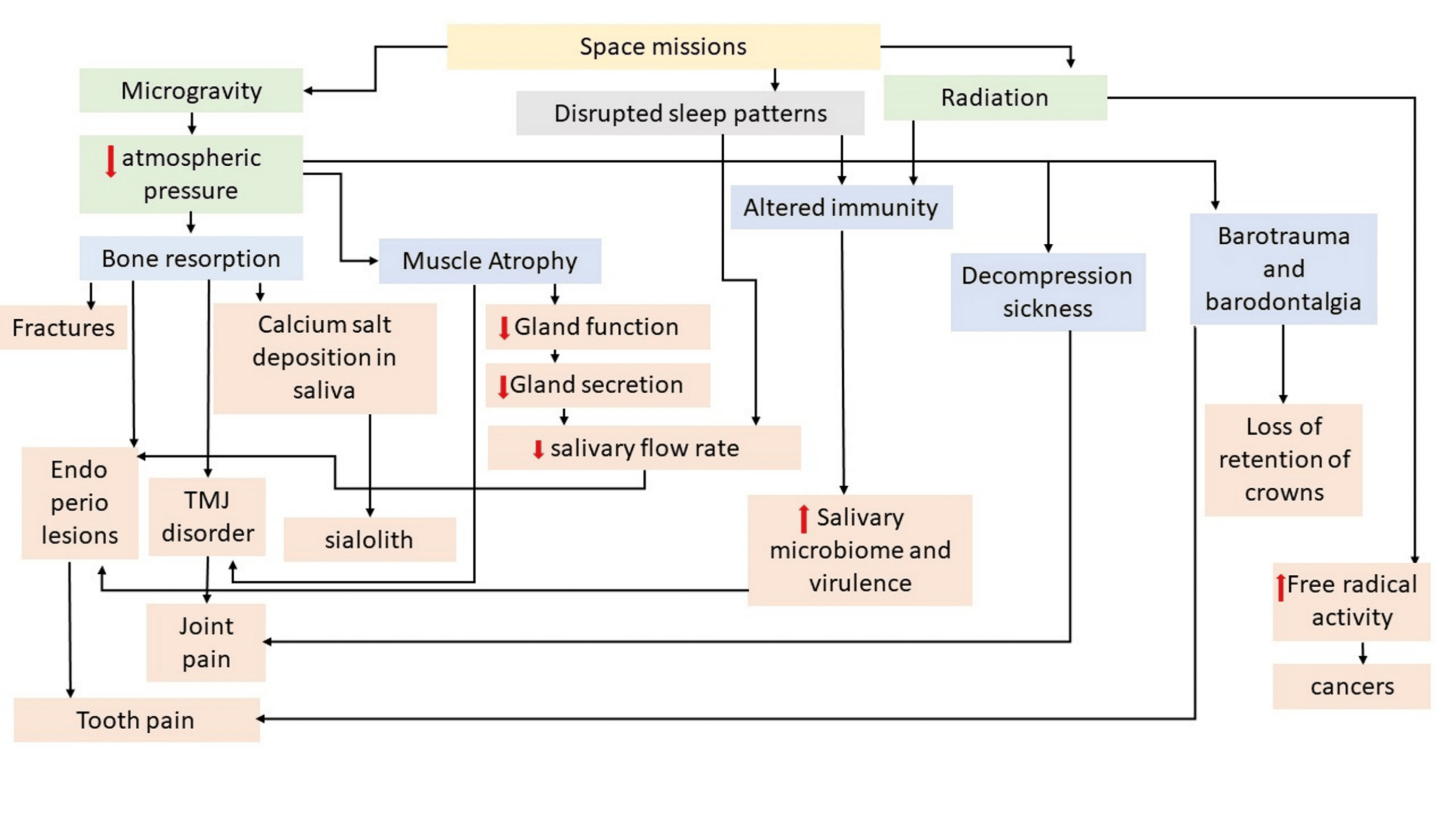
Overview of oral health in space: vulnerability to space-induced changes The figure is the author's creation.

The alterations in the environmental and physiological factors experienced in space lead to the development of a range of oral health issues (Figure 1). 

Endo-Perio Lesions

Alterations in bone density and mineral content, variations in salivary flow rate and composition, changes in immune functions, and modifications in the oral microbiome can synergistically contribute to various endodontic and periodontic lesions in space. Additional factors like suboptimal oral hygiene, elevated stress levels, and onset of fatigue can exacerbate these lesions. In the space environment, astronauts are administered pure oxygen, which has the potential to cause corrosion of amalgam dental fillings. Psychological factors, altered sleep patterns, and several other factors combine to trigger teeth grinding and clenching (bruxism), ultimately causing attrition and abrasion of tooth surfaces and compromising the integrity of dental restoration [17].

Dental Barotrauma

Barotrauma is a condition that can affect the tissues due to variations in pressure between the body's gas-containing spaces and the surrounding atmosphere, often occurring during high-altitude travel. This concept relies on Boyles law, which establishes that under constant temperature conditions, the volume of gas is inversely related to surrounding pressure [4]. Fluctuations in external barometric pressure can lead to breakage of teeth or dental restorations. This phenomenon is known as dental barotrauma. When the tooth that has been restored contains a tiny void (gas pocket) within the restoration, this void can either expand or contract in response to the fluctuations in surrounding pressure. As a consequence, it generates enough pressure that could lead to a fracture of the tooth or restoration [17]. In the absence of gravity, the reduced atmospheric pressure may trigger the expansion of trapped gas beneath previously compromised restorations, or recurrent carious lesions may contribute to this tooth explosion phenomenon. This process is medically referred to as odontocrexsis (tooth + explode). According to Calder and Ramsey, subpar dental restorations, whether or not accompanied by cavities, pose a risk of dental injury [5].

Barodontalgia

Barodontalgia is a dental pain evoked by a change in barometric pressure in an otherwise asymptomatic tooth. Barodontalgia is a symptom, not a pathologic illness in and of itself. Barodontalgia is usually a flare-up of preexisting sub-clinical oral-maxillofacial disease caused by a change in barometric pressure. Dental caries, faulty tooth restoration, pulpitis, pulp necrosis, apical periodontitis (jawbone cyst and granuloma), periodontal pockets, impacted teeth, and mucus retention cysts have all been identified as probable causes of barodontalgia [29]. There are two types of barodontalgia: 'direct' barodontalgia, which is related to pulp/periapical reasons, and 'indirect' barodontalgia, which is induced by barotitis/barosinusitis. It can be caused in two ways- either due to the creation of pressure changes in the tooth, caused by carious activities, or defects in it. In dental abscesses, deep lesions, and unlined restorations, gas pockets expand under reduced air pressure, causing pressure buildup, pain, discomfort, and impaired organ function. It can also occur during ascent, as the pressure reduces, leading to the dissolution of gases in the blood vessels, due to which bubbles enter the pulp [30].

Temporomandibular Joint Disorders (TMDs)

In a state of weightlessness, the decrease in mechanical force on skeletal structures and articulating surfaces leads to skeletal porosity and ultimate bone density reduction. This results in bone weakness and a predisposition to fracture and weakening of skeletal muscles. Microgravity conditions affect the body's circadian rhythm and physiological changes, causing psychological stress and sleep disturbances. The impact of this stress influences the construction of the temporomandibular joint and the overall bone mineral mass reduction. TMDs have complex origins, including sleep anomalies and stress induced by microgravity conditions. Increased levels of psychological distress are observed among the TMJ and skeletal muscle groups, and psychological domains influence this distress. TMJ pain tends to be less severe than muscle pain. TMDs are associated with the disruption in cortisol regulation and melatonin secretion. Probably, the heightened activation of the stress hormone system due to conscious pain perception provides an explanation for these phenomena [5]. Long-term stressful conditions and mental illnesses cause occlusal parafunction, like bruxism, which might lead to temporomandibular disorders. They can also result in masticatory muscle pain and significant dental and periodontal damage due to the physical strains they are subjected to.

Infections

A weakened immune system paves the way for a surge in both the quantity and virulence of microorganisms in the microgravity environment, ultimately leading to infections during space missions. The entire Apollo 11 crew exhibited symptoms resembling a viral upper respiratory infection. Following the Apollo 13 mission, the implementation of regular pre-flight quarantines has notably curbed the prevalence of infectious diseases in space travel. Consequently, minor infections like boils, stye formation, and gum infections have been predominant issues reported in later missions [21].

Trauma

Traumatic injuries pose a significant threat to human space exploration, primarily due to its expected frequency and the substantial consequences it can have on crew well-being and mission success. Penetrating trauma is a potential risk during extravehicular activities and could lead to rapid decompression and fatality. Blunt trauma might result in potential damage to organs and hemorrhagic shock, which is the primary focus of concerns in cases of extraterrestrial trauma. Furthermore, the physiological changes affecting bone density may render jaw bones more fragile and susceptible to fractures, increasing the likelihood of jaw and dental injuries [21].

Countermeasures and developments

Dental emergencies might risk the mission, and dental treatments in space are challenging. Hence, preventing dental disorders and maintaining oral health must be prioritized. All astronauts, whether assigned to crew or not, are informed of the benefits of a healthy diet and encouraged to practice thorough oral hygiene. Periodic checkups are carried out on crew members. NASA upholds rigorous criteria for selecting, retaining, and assessing the oral cavity of the designated crew members assigned to a particular space mission, and a stern clinical agenda is pursued [6]. They undergo clinical and radiographic oral examinations annually, including bitewing and panoramic radiographs [17].

Astronauts are grouped into three categories according to their oral health [17], as shown in Table 1.

**Table 1:** Astronaut oral health classification

Astronaut class	Description
Class I	Good oral health; no need for dental treatment or re-evaluation within 12 months
Class II	Some oral conditions present, but no imminent dental emergency expected within 12 months if left untreated
Class III	Oral conditions present, immediate dental treatment required to prevent a dental emergency within 12 months

All astronauts are expected to retain a minimum Class II status, and only astronauts with Class I status prior to launch are considered for the International Space Station [17].

Half a year prior to take-off, astronauts go through a comprehensive examination. If dental interventions are found to be essential, these procedures are scheduled and completed three months before take-off to reduce the risk of potential dental issues during the mission [6].

During flight, remote diagnostic systems perform routine oral health checkups. Photographs of astronauts' oral cavity would be captured with an oral camera and transmitted to the ground control room. For inflight dental emergencies, medical equipment and instruments for various potential medical procedures are available onboard the International Space Station, with detailed instructions. The Dental Pack/manual for astronauts (Table 2) in the space environment is composed of handling the following procedures (NASA Medical Checklist 2001): 1) Crown Replacement, 2) Total Avulsion / Complete Tooth Loss, 3) Exposed Pulp, 4) Injection Technique, 5) Temporary Filling, 6) Tooth Extraction, and 7) Toothache. Dental treatment protocols in space are modified to the restrictions of using water and sharpening tools onboard the spacecraft [7].

**Table 2:** Space mission dental checklist

Checklist	Description
Crown replacement	Procedures for replacing dental crowns in space, considering limitations in water and tools onboard spacecraft
Tooth avulsion	Protocol for addressing total tooth loss in the space environment
Exposed pulp	Guidelines for handling exposed pulp in dental emergencies during space mission
Injection technique	Procedures for administering dental injections under space constraints
Temporary filling	Protocol for temporary dental fillings in space, considering available resources
Tooth extraction	Instructions for tooth extraction in space, where traditional methods may not be feasible
Tooth ache	Management of toothache and dental pain during space missions according to the manual

Carious lesions that have ceased progression (arrested lesions) pose less threat on earth, as they remain inactive, and the likelihood of reaching pulp is unlikely. However, in an environment with changing pressure, such lesions carry risks and should be addressed [4].

In case of incidence of dental caries or dislodgement of fillings in ISS missions, cavit (a temporary restorative material) can be used, but it acts only as a short-term solution, not suitable for more extended missions due to its limited durability, typically lasting only two weeks to one month [17].

In cases when individuals on Earth would typically be advised for direct/indirect pulp therapy, those preparing for spaceflights should be recommended for an endodontic procedure instead. This distinction is due to the potential impact of Boyle's law, where any void between the pulp capping material and pulp could undergo volumetric expansions in microgravity conditions. Hence, it is crucial to ensure no pulp exposure following the removal of caries, and in case of exposure of pulp, complete root canal treatment must be done prior to spaceflights as incomplete access openings lead to further complications [17].

In the context of prosthesis cementation, the choice of luting cement significantly affects the retention of crowns in space. The presence of continuous pressure fluctuation within tiny air bubbles in the cement layer beneath the crowns can result in reduced crown retention. Resin cement outperformed zinc phosphate and glass ionomer cement (GIC) in terms of retention and microleakage prevention due to porosities incorporated at the time of manipulation of zinc phosphate and GIC. These porosities may cause barotrauma, resulting in a weakened cement layer [17]. Decreased barometric pressure may also lead to compromised removable denture retention. Therefore, prosthesis supported by implants is the superior choice [4].

Dentists should investigate oroantral communication during extraction of the posterior upper tooth, which can pose a potential risk when exposed to barometric pressures [4]. Recently, there have been advancements in bandages and dressings that are effective in controlling hemorrhage. In addition to conventional gauze, tissue sealant dressings, similar to fibrin-based adhesives in composition, lead to significantly reduced blood loss [17].

In the world of microgravity, ensuring the safety of both the patient and clinician becomes paramount. The abundance of weightless, non-sterile particles floating about in the cabin makes it imperative to meticulously plan how to maintain the sterility of the surgical environment and protect against wound contamination. In this unique setting, it is crucial to establish a reliable restraint system. Sponges and suction have proven effective in averting contamination of the cabin resulting from bleeding, except in cases of arterial bleeding, which presents a more complex challenge [23].

Future Prospects

The aerospace dentistry future is highly influenced by the convergence of technological innovations and an understanding of the consequences of space on oral health. Anticipated advancements include 3D printing for the on-demand fabrication of customized dental prostheses and devices during space missions. Nanotechnology will drive the development of innovative dental materials capable of withstanding radiation exposure and maintaining optimal performance in the challenging space environment. Advances in tele-dentistry, incorporating AI algorithms and wearable devices to monitor astronauts during missions continuously will allow real-time consultation with dental professionals on Earth. Additionally, potential innovative approaches such as personalized medicine and the incorporation of artificial gravity should be researched.

## Conclusions

The exploration of space presents a unique set of challenges, and the field of dentistry is no exception. This comprehensive literature review highlights space travel's multifaceted and complex implications on oral health. Space missions' extended duration and remoteness significantly escalate the potential for dental emergencies, necessitating a strategic focus on pre-flight and in-flight preventive measures and preparing crew members to manage dental incidents effectively. Yet, it is evident that this niche within dentistry remains relatively underexplored, opening up a vast landscape for novel research and innovation. As the vision of human space exploration beyond Earth’s orbit continues to evolve, investing in comprehensive research and advancement in dentistry in space is imperative. Proactive measures to address the oral health challenges in space will contribute to the successful execution of prolonged missions, enable astronauts to perform at their best, and pave the way for safe and sustainable human presence in the final frontier.

## References

[1] Krittanawong C, Singh NK, Scheuring RA (2022). Human health during space travel: state-of-the-art review. Cells.

[2] Wolfe JW, Rummel JD (1992). Long-term effects of microgravity and possible countermeasures. Adv Space Res.

[3] Arone A, Ivaldi T, Loganovsky K, Palermo S, Parra E, Flamini W, Marazziti D (2021). The burden of space exploration on the mental health of astronauts: a narrative review. Clin Neuropsychiatry.

[4] Bansal P, Nikhil V, Ali S, Mishra P, Bharatiya R (2018). Space and aeronautical dentistry: a review. Indian J Pharm Pharmacol.

[5] Stevens M, Keyhan SO, Ghasemi S (2020). Does microgravity effect on oral and maxillofacial region?. Int J Astrobiol.

[6] Rai B, Kaur J (2011). The history and importance of aeronautic dentistry. J Oral Sci.

[7] Häuplik-Meusburger S, Lotzmann U, Meusburger H (2016). Dental treatment during a human Mars Mission with remote support and advanced technology. ICES.

[8] (2013). Review of Spaceflight Dental Emergencies. https://ntrs.nasa.gov/citations/20120002807.

[9] Brown LR, Frome WJ, Handler S, Wheatcroft MG, Rider LJ (1977). Skylab oral health studies. Biomedical Results from Skylab (NASA SP-377).

[10] Man J, Graham T, Squires-Donelly G, Laslett AL (2022). The effects of microgravity on bone structure and function. NPJ Microgravity.

[11] Chitkara N, Garg A, Mittal R (2017). Exploring astronautical dentistry: a review. Int Health Res J.

[12] Sibonga JD, Evans HJ, Sung HG (2007). Recovery of spaceflight-induced bone loss: bone mineral density after long-duration missions as fitted with an exponential function. Bone.

[13] Caillot-Augusseau A, Lafage-Proust MH, Soler C (1998). Bone formation and resorption biological markers in cosmonauts during and after a 180-day space flight - Euromir 95. Clin Chem.

[14] Rai B, Kaur J, Catalina M (2010). Bone mineral density, bone mineral content, gingival crevicular fluid (matrix metalloproteinases, cathepsin K, osteocalcin), and salivary and serum osteocalcin levels in human mandible and alveolar bone under conditions of simulated microgravity. J Oral Sci.

[15] Sun H, Zhou Q, Qiao P, Zhu D, Xin B, Wu B, Tang C (2022). Short-term head-down bed rest microgravity simulation alters salivary microbiome in young healthy men. Front Microbiol.

[16] Rai B, Kaur J, Foing BH (2011). Evaluation by an aeronautic dentist on the adverse effects of a six-week period of microgravity on the oral cavity. Int J Dent.

[17] Goyal A, Malhotra P, Bansal P, Arora V, Arora P, Singh R (2015). Mission mars: a dentist’s perspective. J Br Interplanet Soc.

[18] Agha NH, Baker FL, Kunz HE (2020). Salivary antimicrobial proteins and stress biomarkers are elevated during a 6-month mission to the International Space Station. J Appl Physiol.

[19] Urbaniak C, Lorenzi H, Thissen J (2020). The influence of spaceflight on the astronaut salivary microbiome and the search for a microbiome biomarker for viral reactivation. Microbiome.

[20] Rooney BV, Crucian BE, Pierson DL, Laudenslager ML, Mehta SK (2019). Herpes virus reactivation in astronauts during spaceflight and its application on Earth. Front Microbiol.

[21] Stewart LH, Trunkey D, Rebagliati GS (2007). Emergency medicine in space. J Emerg Med.

[22] Philippou A, Minozzo FC, Spinazzola JM, Smith LR, Lei H, Rassier DE, Barton ER (2015). Masticatory muscles of mouse do not undergo atrophy in space. FASEB J.

[23] Hodkinson PD, Anderton RA, Posselt BN, Fong KJ (2017). An overview of space medicine. Br J Anaesth.

[24] Blue RS, Bayuse TM, Daniels VR, Wotring VE, Suresh R, Mulcahy RA, Antonsen EL (2019). Supplying a pharmacy for NASA exploration spaceflight: challenges and current understanding. NPJ Microgravity.

[25] Putcha L, Berens K, Marshburn T, Ortega HJ, Billica RD (1999). Pharmaceutical use by U.S. astronauts on space shuttle missions. Aviat Space Environ Med.

[26] Barger LK, Flynn-Evans EE, Kubey A (2014). Prevalence of sleep deficiency and use of hypnotic drugs in astronauts before, during, and after spaceflight: an observational study. Lancet Neurol.

[27] Idkaidek N, Al-Ghazawi A (2019). Effect of flying at high altitude on early exposure of paracetamol in humans. Drug Res (Stuttg).

[28] Dello RC, Bandiera T, Monici M, Surdo L, Yip VL, Wotring V, Morbidelli L (2022). Physiological adaptations affecting drug pharmacokinetics in space: what do we really know? A critical review of the literature. Br J Pharmacol.

[29] Lakshmi Lakshmi, Sakthi DS (2014). Aviation dentistry. J Clin Diagn Res.

[30] Zadik Y (2009). Aviation dentistry: current concepts and practice. Br Dent J.

